# The value of hope: development and validation of a contextual measure of hope among people living with HIV in urban Tanzania a mixed methods exploratory sequential study

**DOI:** 10.1186/s40359-020-0376-y

**Published:** 2020-01-29

**Authors:** Hellen Siril, Mary C. Smith Fawzi, Jim Todd, Magreat Somba, Anna Kaale, Anna Minja, Japhet Killewo, Ferdinand Mugusi, Sylvia F. Kaaya

**Affiliations:** 1 Department of Psychiatry and Mental Health, Muhimbili, University of Health and Allied Sciences (MUHAS), P.O. Box 65001, Dar es Salaam, Tanzania; 2 Department of Global Health and Social Medicine, Harvard Medical School, 641 Huntington Ave, Boston, MA USA; 3 Department of Population Health, London School of Hygiene and Tropical Medicine, London, UK; 4 Africa Academy for Public Health (AAPH), Plot # 802 Mwai Kibaki road, Dar es Salaam, Tanzania; 5 Muhimbili University of Health and Allied Sciences (MUHAS), P.O. Box 65001, Dar es Salaam, Upanga Tanzania; 6 Department of Internal Medicine P.O. Box 65001, Muhimbili University of Health and Allied Sciences (MUHAS), Dar es Salaam, Tanzania

**Keywords:** Hope, Scale development, Validation, HIV, PLH, *NAMWEZA*, Dar Es Salaam

## Abstract

**Background:**

Hope or hopefulness enhances coping and improves quality of life in persons with chronic or incurable illnesses. Lack of hope is associated with depression and anxiety, which impact negatively on quality of life. In Tanzania, where HIV prevalence is high, the rates of depression and anxiety are over four times higher among people living with HIV (PLH) compared to persons not infected and contribute annual mortality among PLH. Tanzania has a shortage of human resources for mental health, limiting access to mental health care. Evidence-based psychosocial interventions can complement existing services and improve access to quality mental health services in the midst of human resource shortages. Facilitating hope can be a critical element of non-pharmacological interventions which are underutilized, partly due to limited awareness and lack of hope measures, adapted to accommodate cultural context and perspectives of PLH. To address this gap, we developed and validated a local hope measure among PLH in Tanzania.

**Methods:**

Two-phased mixed methods exploratory sequential study among PLH. Phase I was Hope-related items identification using deductive, inductive approaches and piloting. Phase II was an evaluation of psychometric properties at baseline and 24 months. Classical test theory, exploratory, confirmatory factor analysis (CFA) were used.

**Results:**

Among 722 PLH, 59% were women, mean age was 39.3 years, and majority had primary school level of education. A total of 40 hope items were reduced to 10 in a three-factor solution, explaining 69% of variance at baseline, and 93% at follow-up. Internal consistency Cronbach's alpha was 0.869 at baseline and 0.958 at follow-up. The three-factor solution depicted: positive affect; cognition of effectiveness of HIV care; and goals/plans/ future optimism. Test-retest reliability was good (*r* = 0.797) and a number of indices were positive for CFA model fit, including Comparative Fit Index of 0.984.

**Conclusion:**

The developed local hope scale had good internal reliability, validity, and its dimensionality was confirmed against expectations. The fewer items for hope assessment argue well for its use in busy clinical settings to improve HIV care in Tanzania. Hope in this setting could be more than cognitive goal thinking, pathway and motivation warranting more research.

**Trial registration:**

The intervention was registered in USA ClinicalTrials.gov on September 26, 2012, Registration number: NCT01693458.

## Background

Hope has multiple definitions including a feeling of expectation and desire for a particular thing to happen characterized by having a goal/desire, agency (inner force to act) and plans on how to achieve the goals [1, 2]. Other definitions include a perceived ability to produce pathways to achieve desired goals and to motivate oneself to use those pathways [3]; a positive psychosocial strength [1]. Hope is more than optimism [4]; psychologists have in recent years differentiated hope and optimism, by emphasizing hope as a positive future expectation in which one must have a role to play in order to establish it. Optimism is having a positive future expectation without necessarily playing any role to establish it [5].

Hope is also shaped by culture and social structural issues of the population where an individual exists. Culture values including religious faith, family unit, and honor can act as a source of resilience, driving social aspirations, motivations, self-respect and dignity [6–9]. However social structure inequalities can impede realization of dreams and one’s cultural expectation thus a source of stress frustration and hopelessness. These include poverty, social injustice, war crises and displacements, ineffective governance etc. [7–10]. Other researchers also add that social hope comes from an individual ability to perceive the existing social mobility (possibilities that life can offer) and its opposite or hopelessness is a sense of entrapment or a sense of having nowhere to go and not poverty [11]. The struggle to maintain culturally acceptable standards in the midst of structural limitations can be a source of distress and lack of hope. Cultural norms and values have thus been described as “a double aged sword” which can bring social functioning or social violence and thus distress [12].

Interventions that aim to increase hope have been reported across the globe to help patients cope with difficult situations such as living with chronic or incurable disease, cope with the process of aging, adherence to prolonged care procedures and daily medications [13–16] as well as reducing depressive, anxiety symptoms and increasing healthier lifestyles [17]. Hope has a negative association with depression, anxiety, mixed depression and anxiety and less specific psychological distress [18, 19] and is frequently applied for managing hopelessness and preventing mild to moderate mood disorders [20–23]. Hope is also a mediator of resilience, well-being [24], and in general population health being hopeful contributes to reductions in all-cause mortality [25]. With positive future optimism, support, and a perceived role to play, hope is a core dimension of recovery from mental illness and substance abuse [26, 27]. Among PLH, hope increases coping [28, 29], adherence to HIV care and medications [30] thus potentially improving ART treatment outcomes. Hopelessness, on the other hand, is associated with increased risk of anxiety or depressive disorders [31–35], predicts an increase in depression-anxiety co-morbidity [36] and suicidal behaviors [37, 38]. Increased hopelessness is a contributing factor to increased engagement in HIV-related risk behaviors [39, 40]. Focused interventions that impact positively on hope would thus add an important component to HIV prevention, care and treatment services.

Although studies indicate integrating care for some of the common mental health disorders including depression, alcohol use disorders and anxiety could improve antiretroviral (ART) treatment outcomes [41] among PLH, there is still limited access to quality mental health services within HIV care and treatment services [42, 43]. While in part, this could be a consequence of an existing crisis of human resource for health with lack of sufficient trained mental health care professionals, it is important that they are recognized and managed by general health care providers with an option to refer severe cases [44]. Increasing hope is a non-pharmacological approach that could alleviate hopelessness and mood disorders of mild to moderate forms. However, there is limited use and integration of hope concepts and related non-pharmacological therapies in Tanzania and other African countries. This is in part due to the lack of relevant measures of hope developed for use in African populations living in SSA [28, 45, 46]. Almost all the tools for measuring hope were adopted from western cultural settings and most of them have not been validated in African settings. An Unpublished pilot study conducted shortly before this study that utilized the commonly used hope measure of Snyder hope scale, to measure hope among PLH in Dar es Salaam Tanzania revealed Cronbach’s alpha internal consistence of 60 which is way below the recommended cut offs of 70 and above [47]. Another study conducted in Tanzania that utilized the same scale to measure hope among women in microfinancing groups, reported that the women scored very high and many reached very near to the maximum attainable scores [48]. The same study also showed that the Snyder scale had poor discrimination and strong evidence of acquiescence response bias. This indicates the need to further explore potential hope scales developed in African cultural settings.

In order to address these gap, we developed and validated a measure of hope that is based in high HIV prevalence setting of urban Africa.

### Study objective

The aim of this study was to develop and validate a local measure for hope among PLH in a high HIV prevalence and low-income setting that will reflect understandings of hope among PLH and consider the relevant terms used to express hope in this socio-cultural context [49]. This was an adjunct study done to complement the main larger *NAMWEZA* study.

## Methods

### Study setting

The study was conducted in Dar es Salaam, the largest city in Tanzania, with a population of 4.5 million, and just over half (51%) residing in informal settlements [50]. The city has the third highest prevalence of HIV in the country of 6.2% against a national prevalence of 5.2% [45]. Annual mortality among PLH accessing antiretroviral (ART) medication is 13.1% [51], and depressive disorders are among mortality contributing factors [51–54]. Rates of clinically significant depressive symptoms in PLH range from 36 to 56%. PLH with moderate to severe depression during ART initiation have a mortality rate that is nearly twice as high compared to those who are not depressed [41]. Phase I was done in 8 large HIV care and Treatment Clinics (CTCs). The CTCs are outpatient clinics available in all government health care facilities which offer health care services exclusively for PLH. The clinics run from Monday to Friday for adults while Saturdays are for pediatric and adolescents. Phase I was conducted 8 large CTCs from the 3 districts of Dar es Salaam. The large CTCs were defined as CTCs that had accumulated enrolled over 5000 PLH before the study began. The CTCs from each district were selected proportionately meaning the district with more CTCs contributed more CTCs to participate in this study. Four CTCs we selected from Ilala district, 2 from Temeke and 2 from Kinondoni district. Phase II was done at one large CTC in the largest district of Kinondoni, Dar es Salaam, which was by the time the study was starting the highly populated district in Dar es Salaam over 50% of the city inhabitants.

### Study design

We conducted a two-phase mixed method exploratory sequential study in three phases [55] with 722 PLH. Phase I included 10 focus group discussions (FGDs) and 8 in-depth interviews with a total of 78 PLH, pile sorting and expert rating which identified hope domains and potential questions which were subsequently piloted them among 318 PLH.

Phase II was a validation phase where the local hope items from the pilot were further tested through a longitudinal sample of 326 PLH who were participants of the larger psychosocial support structured group intervention called *NAMWEZA* [56], using baseline and 24 months follow-up data.

### Description of *NAMWEZA* intervention

#### NAMWEZA

The term comprises truncated Kiswahili words (***NA****A****M*** *TUNA****WEZA*** meaning Yes, together we can!). Is the brand name of a strength-based intervention that utilizes Appreciative Inquiry strategies to motivate participants towards being more positive in building better relationships and in developing and implement individual future life plans. Although this approach is more commonly used in business and other settings with focus on fostering organizational change, it has been used recently within family and community initiatives as well as health interventions [57, 58]. As used in *NAMWEZA*, the Appreciative Inquiry Approach involved searching for the best in people, and the world around them through a systematic discovery of individual strengths, what is going well in ‘life’ and existing support systems instead of searching for what is not working [59]. The Appreciative Inquiry Approach is linked with a positive psychology theoretical framework since it relates to exploring the development of hope and future-mindedness and focusing on self-efficacy and an affirmative perspective of the future [60]. The initial curriculum of *NAMWEZA* was created as a structured, manualized, and in a closed psychosocial group’s format for delivery by trained peers. It emphasised on increasing awareness of one’s own and others inherent abilities and existing social supports; and facilitating effective communication, problem solving, goal setting, and future planning skills. Participants learnt to dream about positive futures, and question in depth the steps needed to attain this future that informed goal setting and action planning. In addition, *NAMWEZA* was implemented within a Stepping Stones Intervention framework including components of experiential learning, real life practice of skills and feed-back as well as understanding and challenging cultural barriers to change [61–63]. These additions aimed to allow the *NAMWEZA* strength-based approach to have higher contextual relevance for piloting as an evidence-based structured psychosocial support intervention delivered in group format, for HIV prevention in PLH, with focus on facilitating hope, reducing risk behaviours and addressing other structural drivers of HIV transmission.

### Characteristics of study participants

The study population included adults living with HIV/AIDS;

#### Eligibility criteria

Phase I (Focus group discussions (FGDs), In-depth interviews (IDIs) and a pilot survey): This phase included adult; 18 years of age and above, living with HIV/AIDS, and enrolled for care the selected CTCs, on antiretroviral (ART) medications for at least 6 months, and agrees to participate and provides a signed informed consent. In addition to phase I participants of the IDIs were required to have had a recent (past 4 weeks) diagnosis of depression or having depression documented in their patient’s clinical records/files or indicated as a reason for lost to follow up or missed clinic visits, in order to for them to be able to provide more depth of information on contracts that arose from FGDs that needed more information based personal experiences on both hope and hopelessness and depression. The other inclusion criteria as indicated in for the phase I above applied for phase II. In addition participants of the phase II were the participants of the larger *NAMWEZA* study and they were included if they were living within the catchment area of Kinondoni district at the time of enrollment and planning to remain living in Kinondoni for a minimum of 2 years and had been on ART for at least 3 months.

#### Exclusion criteria

For both phase I and II, people who were less than 18 years of age, not HIV infected, not enrolled for care in the selected CTCs, not on ART medication, and not being well enough to attend the study interviews and intervention sessions were excluded. Participants who were not living within the catchment area of the former Kinondoni district and those not planning to remain in Kinondoni district after enrollment were excluded from the phase II.

#### Primary outcomes

The primary outcomes included: developed initial draft of local measures of hope, initial psychometric properties of the local hope measures and validated local hope measures among PLH in Tanzania.

### Phase 1: local hope items identification, content validity and piloting

#### Identification of potential questions and domains of the local hope scale

We combined both deductive and inductive approaches to identify potential items to be included in local hope scale. An extensive literature review to understand existing knowledge about the concept of hope and its domains [1, 2, 4, 64–66] followed by an inductive approach of generating items that could measure hope from the emerging themes of the narrative data from participating PLH. The items were then grouped into identified initial domains of hope. The qualitative narrative data were based on findings from a previously published study among 78 PLH participating in 10 focus group discussions (FGDs) and 8 in-depth interviews (IDIs). The participants were selected based on the eligibility criteria listed above. Purposive sampling was used to select the FGD and IDI participants and in addition, snow ball approach was used in CTCs which we could not easily get the IDI participants. The data for the FGDs and IDIs was collected from February to June 2012. More details are available in our previously published study [49]. The data for phase I pilot survey were collected from June 2012 to early August 2012. The data were reviewed by the study team members (local nurses, social workers and physicians) to individually identify items describing hope. Joint discussions were held and consensus reached on 40 items that could be defined as having attributions of hope (see A2 and A4 of Additional file 1). These items included those that did and did not perfectly map on the hope domains previously identified from the literature review, including those that were locally derived. The team also placed narrative data emergent items into three theoretical domains: depicting collaborative/influence of other people (12 items); affective /spiritual positive emotions such as feeling uplifted, comforted, being secure and at peace (13 items); and a cognitive dimension including optimism and positive future anticipations/coping (15 items) (See A3 of Additional file 1).

#### Content validity of the identified hope scale items

The list of 40 items were reviewed further by a target group of PLH and a multidisciplinary panel of experts including one psychiatrist, one mental health researcher, three social scientists, two public health and HIV health care specialists, 1 postgraduate student in the mental health department. The panel scrutinized the items and the domains for correct definitions, relevance, ambiguity, and what hope meant to the informants. This resulted into modifying and expanding to include more specific domains into 6 domains including; positive emotions, current and future fear of living with HIV, satisfaction with HIV care/positive attitude towards HIV and care, planning and positive future expectations (A 3 of Additional file 1) The target population review was done by a group of ten PLH who provided feedback on each item, including its clarity and appropriateness as an attribute of hope from their perspective (if the items really reflected what PLH perceived as hope). Based on this we edited the grammar and word choice in the Swahili language rephrased some of the items.

The list was then sent to two experts psychiatrists recognized by having published work on depression, stress disorders or hope, having a reputation as skilled clinicians working within the Tanzanian mental health and HIV areas who were part the *NAMWEZA* study. The experts, HIV clinic staff and graduate students were asked to review and rate each of the local idioms on the list in a scale of 1 to 5 on the extent to which they thought the item characterized local experiences of hope, as 1 = not at all characteristic, 2 = somewhat characteristic, 3 = no sure if characteristic or not characteristic (neutral), 4 = characteristic and 5 = very characteristic.

**Fig. 1:**
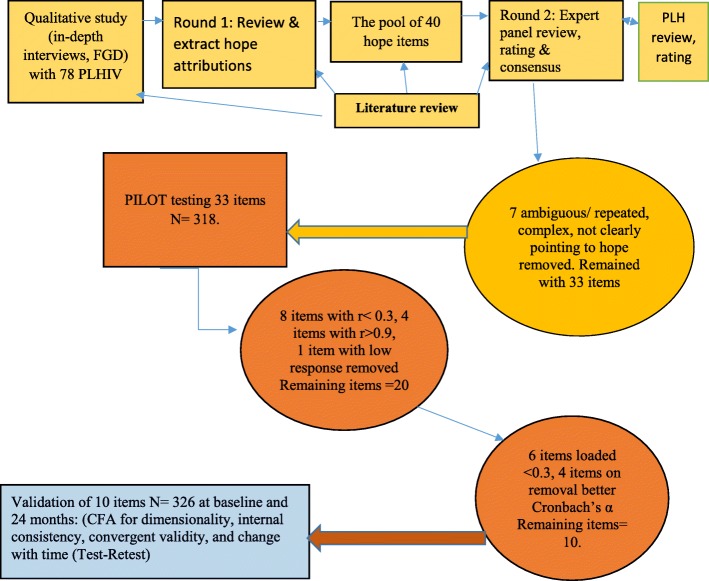
Flow diagram summarizing the procedures used to develop the local hope scale

This was followed by a pile sorting and rating by the 10 PLH who had provided inputs earlier whereby a pile of cards with different idioms of hope were presented to them by having the study RA read the cards aloud to allow more clarity to the participants and to help those that were illiterate to understand. Each participant, privately placed cards in any of the boxes each labeled with 1 of the 6 domains of hope identified and also wrote and added any description of hope they felt was different from what was in the cards*.* The PLH also rated the items placed in each box using a visual and verbal analog Likert scales that allowed the participants to rate the items in each box from 1 to 5; 1 = not at all characteristic, 2 = somewhat characteristic, 3 = sure if characteristic or not characteristic, 4 = characteristic, 5 = very characteristic, through placing a circle on a visual and verbal analog scale the extent to which they felt that the idiom fitted the selected domain on each of the cards and for each of the domains to allow for possible overlap in expressions used for the concepts of interest. The ratings were analyzed systematically. This process resulted into a removal of 7 items (Fig. 1) that were felt to be ambiguous or complex or not attributed hope and generation of the final list of the most frequent items on the PLH’s lists were assessed with those generated by experts and health care providers of items for measuring hope that was then piloted among 318 PLH at 8 large CTCs in Dar es Salaam.

#### Pilot testing and initial psychometric properties

The remaining 33 items were phrased as opinion statements with a four-option Likert scale response format (definitely false = 1, somewhat false = 2, somewhat true = 3, and definitely true = 4), and piloted among 318 PLH.

#### Sampling and data collection for pilot testing

Our sample size for the pilot survey was based on evidence and recommended minimum sample for factor analysis which is 2–20 times the number of variables [67]. We had 33 variables and we iteratively chose our sample fit by looking at the Kaiser-Meyer-Olkin measure of sampling adequacy which was 0.83 thus above the commonly recommended value of 0.6 for factor analysis and Bartlett’s test of sphericity, which was significant for the hope scale items (χ2 (253) = 24,960.00, *p* < 0.0001) when our sample was 300. To allow for potential non-response, and missing data we randomly selected and enrolled 318 PLH from 10 large HIV care and treatment clinics who agreed to participate and completed the pilot questionnaire with a response rate of 100%.

#### Pilot testing procedures

The 33 hope items derived from the qualitative data were piloted among 318 pilot survey participants PLH. The aim of the pilot was to get a first glimpse of the initial responses to the local hope scale items, to explore items that may have been difficult to understand and make rephrasing or removal decisions, as well as the initial psychometric properties of the scale before phase II of validating the scale in a baseline and follow-up (occurring 24 months after baseline) surveys nested within the *NAMWEZA* effects evaluation survey [47] started. This entailed examining item response rates and item-item bivariate correlations to identify extreme responses and unanswered items. Items that had 90% or higher non-response rates were removed, with the assumption that participants found them unclear, or redundant. Items with Spearman’s bivariate correlation coefficient(r) < 0.3 or > 0.9 were also removed. This resulted in 20 items for inclusion in the next phase of the analysis.

Hope pilot study data were collected using a paper-based questionnaire administered on a face-to-face interview with eligible PLH done by trained social scientists. The trained study RAs contacted every third person coming for their routine HIV clinic visit in the selected 8 CTCs for eligibility prior to an informed consenting process. This was done in order to allow time for screening and reasonable spacing of participants screened to avoid selecting groups of people who come together who might be peers and may be share similar characteristics and leave out other eligible participants who could be different from the groups. The process continued until the targeted number of participants to be enrolled was reached.

#### Exploratory factor analysis (EFA) and scale reduction

Exploratory Factor Analysis (EFA) was conducted on the 20-items to identify the optimal number of factors or hope constructs that could be extracted by examining eigenvalues or the variance in response that they explained. Factors loading with eigenvalues equal to or greater than one were considered potential to retain. In each specific factor extracted only individual items loading at 0.3 or more were retained. Finally, scale reliability analyses were conducted retaining only those items that improved internal consistency, as assessed using Cronbach’s alpha, without altering the meaning of the extracted factors. In addition, we checked at each step, item-item bivariate correlations to ensure items that had very small (*r* < 0.3) or very high correlation coefficients (*r* > 0.9) were removed. (Fig. 1). These analyses resulted in retaining 10 items of the hope scale which were then subjected to confirmatory factor analyses (CFA) and scale reliability evaluation.

**Fig. 2:**
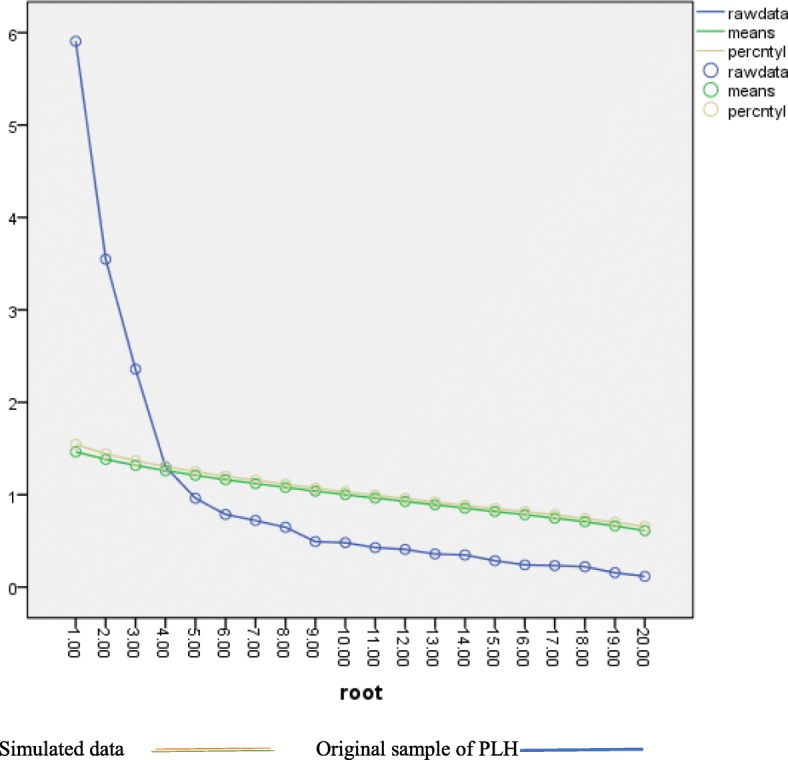
Comparison of study Eigenvalues with simulated Eigenvalues Depicting 3 Factor Solutions during Pilot

Additionally, EFA revealed the potential number of hope dimensions that our data would most likely present by looking at the plot of relative values of eigenvalues for each hope factor (also known as a scree plot) that fell above the plot’s elbow point (a point where the plot becomes horizontal). This captures factors with items that explain substantial levels of variance and is an indication of factors that contribute to the hope scale’s structure. A parallel analysis (Fig. 2) further confirmed the potential number of factors for our data. This is through examination of computer simulations of multiple random datasets which generated simulated eigenvalues that we compared to the actual eigenvalues resulting from our data. The simulated eigenvalues indicate the amount of random variance in the data that would be expected by chance, therefore actual eigenvalues that exceed simulated eigenvalues were considered candidates for retention in the scale’s factor structure. In these analyses, 1000 randomly selected datasets were used to generate simulated eigenvalues [68]. Finally, EFA revealed the internal consistency of the hope scale as measured at different points while reducing the number of items until we reached our final number of items, with the most reasonable meaning and internal consistency that was within an acceptable range (Fig. 2).

### Phase II: Hope scale evaluation

The purpose of phase II analyses was to explore and confirm the post-pilot tested hope scale’s psychometric properties and its structure. Baseline data were collected from August –end of October 2012 while follow up data were completed in January 2014. In addition follow-up data for assessing hope were collected from March to June 2014.

Local hope measures were collected using a paper-based questionnaire while an Audio Computer-Assisted Self-Interview (ACASI) software captured demographic characteristics and other variables of interest for the larger *NAMWEZA* study which were extracted and linked to the local hope measures using unique participants ID.

Descriptive characteristics confirmatory factor analyses were conducted using SPSS statistical software, versions 20 and 23 [69]. The confirmatory factor analysis was done using SPSS 23 with AMOS software plugins.

#### Confirmatory factor analysis (CFA)

This was conducted in order to test for dimensionality of the hope local measures. We hypothesized that the factor structure extracted during baseline survey and at 24 months of follow-up time would be approximately the same [70] and conducted the test of invariance using CFA model fit using the AMOS program [71] to run the analysis and used 4 indices to confirm the model fit: including chi-square test of exact fit; Root Mean Square Error of Approximation (RMSEA ≤0.08); Tucker-Lewis Index (TLI was 0.978); and the Comparative Fit Index (CFI ≥ 0.969).

#### Assessment of hope scale reliability

We defined reliability as a degree of consistency exhibited when the hope measurement is repeated under identical conditions. Internal consistency was tested using Cronbach’s Alpha and value of a larger than 0.75 indicates satisfactory internal consistency for the main scale and subscales. Test-retest reliability was assessed using two subsequent time points at baseline and 24 months of follow-up [70]. The test-retest reliability was used to assess the degree to which participants sum scores were persistent across time. Intraclass correlation coefficient was calculated to estimate test-retest reliability with correlation efficiency higher than 0.5 considered as good reliability.

#### Construct validity

Convergent validity was performed by examining the associations between the hope scale scores and similar constructs using Pearson correlation coefficient. Stronger correlation coefficients indicating support for convergent validity. In addition, the correlations between mean hope and that of constructs from the literature, including knowledge, social support, self-esteem, adherence to clinic visits, clinical parameters including weight and CD4 + T cells that were expected to be positively associated with hope were used as additional tests of convergent validity. We created a mean hope score from the 10 final items ranging from ‘1’ for complete disagreement to ‘4’ for complete agreement with items. This was followed by determining Pearson’s correlation coefficients between the hope scale mean and selected constructs that we expected to be negatively or positively associated with hope. These included: general knowledge about HIV ART medication, depression; stigma; social support; self-esteem; and adherence to medication and clinic visits. For the convergent validity, we hypothesized a positive relationship between mean hope scores and higher general HIV knowledge, knowledge about ART scores; and similarly, to with other positive constructs including high self-esteem and social support, and good adherence to ART and clinic attendance on scheduled visits. In addition, we hypothesized that positive clinical outcomes will be positively correlated with hope scores with the strength of the association providing support for convergent validity.

#### Discriminant validity

Was assessed by examining the relationship between the hope scale mean scores and distinct constructs from the literature that are negatively related to hope. We hypothesized that depression assessed using the PHQ-9, stigma, and the locally derived *msongo* (a local tool for measuring stress) and *sonona (*a local tool for measuring depression would be negatively correlated with the local hope measures. Small and or negative correlation coefficient was used to support discriminant validity.

## Results

### Description of the study sample

A total of 722 PLH participated: 78 from our previous qualitative study (49); 318 PLH in the pilot survey; and 326 PLH participated in the baseline and 24 months’ follow-up surveys. Descriptive characteristics for the study sample at baseline are shown in Table 1. The participants’ age ranged from 18 to 75 years with a mean of 39.3 years (SD = 10.1) and about 59% were women. The mean number of years spent on primary and secondary levels of formal education was 6.8 years, (SD = 0.75), ranging from 2 to 8, and 4.4 years (SD = 1.8) ranging from 2 to 8, respectively. The mean years at college and degree education was 3 years (SD = 1.2), from 1 to 4, and 3.08 years (SD = 2.0) ranging from 1 to 6 respectively. Participants had low general HIV knowledge (mean score was 3.4; SD = 3.9 which was below half of the expected 8 points. and had a mean score for depression falling below the cut-off score of 9 for clinically significant depression (mean 8.0; SD = 5.6; range 0–27. Mean score for internalized HIV related stigma was above average at 3.6 (SD = 0.95; range 1–5). Participants attended at least 1 clinic visit per month with an annual average of 12.4 (SD = 4.6; range 1–38) clinic visits, had moderate scores of ART adherence; 1.7 scores, (SD = 0.3) ranging from 1 to 3, had moderate hemoglobin levels with a mean of (11.2 mmol/L) SD = 2, range from 5 to 16 mmol/L, and low CD4+ T cells, average 338 cells /mm^3^, (SD = 188.6), ranging from 0 to 1212 cells/mm^3^. The mean weight for the participants was 59.9 kg, (SD = 10.8), ranging from 30 kg–105 kg (Table 1).

**Table 1:** Descriptive Characteristics of the Study Participants at Baseline (*N* = 326)

Characteristic	Sample/n (%)	Mean/score (SD)	Range
Age in years		39.0 (10.1)	18–75
Sex: Women	192 (59)		
Employment status			
Employed/self-employed/housewife/student	283 (87)		
Not employed	43 (13)		
Years in School			
Primary School		6.8 (0.8)	2–8
Secondary School		4.4 (1.8)	2–8
Non-degree/Collage		3.0 (1.2)	1–4
Degree/University		3.1 (2.0)	1–6
General HIV knowledge		3.6 (3.9)	1–8
Knowledge about ART		2.8 (0.6)	1–4
Hope		2.6 (0.5)	1–4
Psychosocial health:			
^a^Depression scores		8.0 (5.6)	0–27
Self-esteem		3.1 (0.6)	1–4
Stigma		3.6 (1.0)	1–5
^b^Perceived social support		2.3 (0.5)	1–3
Local scale of stress scores *(msongo)*		18.4 (4.3)	0–20
Local scale of depression scores *(sonona)*		38.1 (6.0)	0–64
Health seeking behavioral			
Adherence to medication		1.7 (0.7)	1–3
Clinic visits		12.4 (4.6)	1–38
Clinical outcomes			
Hemoglobin(g/dl)		11.2 (2.0)	5–16
CD4+ T cell (cells/mm3)		338.6 (188.6)	0–1212
Weight (kg)		59.9 (10.8)	30–105

^a^Depression scores were measured by PHQ9 tool >/=9 is depression. Scores < 9 no mild or no depression

^b^Perceived social support was measured by (name of the scale) scale, whereby 1 = perceived the highest support, 4 perceived no support

### Hope scale development and validation

#### Item selection and reduction

The detailed descriptions from PLH of the meaning and how they experienced hope, as well as our theoretical conceptual framework on which we based to develop our scale is available in our previously published paper [49].

Of the 33 hope scale question items derived from FGDs and IDIs piloted among 318 PLH one item (item number 30) was removed because it had a low response rate where almost 95% of the PLH chose not respond to it, most likely because the item was unclear and participants might have not understood it well. The local hope scale remained with 32 items of which 22 items which had mean scores of 3 and above ranging from 3.0 (SD = 0.96) to 3.53 (SD = 0.78). Ten items had mean scores ranging from 2.0 (SD = 1.90) to 2.97 (SD = 0.93) while only 1 item had a mean score slightly less than two; 1.99 (0.17) (See A5 and 6 of Additional file 1). Over 50% of participants rated 31 items towards hopeful (somewhat true) or very hopeful (definitely true), while 50% of participants responded definitely false to 1 item (A5 of Additional file 1). Eight other items had very low correlation coefficients (*r* < 0.3) while 4 items had very high correlation coefficients (*r* > 0.9) and were also removed leaving the local hope scale with 20 items (Fig. 1).

#### Exploratory factor analyses (EFA) and hope scale reliability

The initial 20 hope scale items when subjected to EFA during the pilot, the derived scree plot elbow-point and parallel analysis suggested the extraction of three factors contributing to most of the variance in factor loadings, indicating a 3-factor solution depicting positive emotions (affect), planning/plans and positive future expectations. The 20-item hope scale items had a very good internal consistency with Cronbach’s alpha 0.81. Through an iterative approach of excluding items that loaded < 0.3 on a single factor, while maintaining an internal consistency of Cronbach’s alpha of 0.750 and above, eight more items that loaded less than 0.3 in individual factors were removed remaining with a 12-item hope scale with an internal consistency of Cronbach’s alpha 0.87. After more reflection and discussions on how meaningful the items were in each dimension, two items were removed from the hope scale without compromising the internal consistency (Cronbach’s alpha, 0.89), retaining 10 items in the hope scale. The three dimensions of this reduced scale explained 69% of the total variance and these dimensions included positive affect, cognition of effectiveness of HIV care, and future optimism, with internal consistencies of 0.84, 0.78 and 0.79, respectively (Table 2).

**Table 2:** Local hope scale items loading and dimensions, at baseline and at 24 months follow up

Hope Scale Item	Baseline (*N* = 326)			Follow up (*N* = 326)		
Dimension 1 = Affective /positive emotions						
I am Happy	0.740			0.802		
I have peace	0.692			0.813		
I feel I have new strength to move on	0.680			0.759		
My heart is cheerful	0.649			0.763		
Dimension 2 = Cognitive positive results of HIV /ART care						
I am satisfied with my progress since I started ARVs		0.749			0.889	
I believe to follow the advice from my counselor, brings me good results for my health		0.729			0.829	
All my needs for HIV care that I need for my HIV condition are all met		0.555			0.796	
Dimension 3 = planning and future optimism						
I can reach/meet my goals			0.712			0.833
I believe in my plans			0.700			0.771
I can live a long life and continue with my routine activities			0.602			0.846
Overall internal consistency of the local hope scale	0.869			0.958		
Internal consistency Cronbach’s Alpha for subscales	0.843	0.778	0.789	0.93	0.90	0.92
Total Variance explained	45%	13%	10%	64%	20%	10%
Total variance explained	69%			93%		

The evaluation results indicated that the intraclass correlation coefficient between baseline and 24 months of follow-up was high and within the acceptable ranges (*r* = 0.797) and thus positive for test-retest reliability.

#### Local hope scale dimensionality

A total of 10 items were subjected to CFA. All 4 indices for CFA model fit were supportive of a three-factor structure; at baseline, the Root Mean Square Error of Approximation (RMSEA) was 0.045, and the Chi-Square test was insignificant (*p* = 0.13) as recommended, the Tucker-Lewis Index (TLI) was 0.977 and the Comparative Fit Index (CFI) was 0.984. When the baseline survey data were compared with the follow-up survey data the test of invariance for the hope scale was confirmed, with most of the parameters indicating model fit; the RMSEA of 0.032 at follow-up was less than the recommended upper limits of 0.08. However, the Tucker-Lewis Index (TLI) was 0.971 which is above the recommended lower limits of 0.96, and the Comparative Fit Index (CFI) was 0.975 which is higher than the recommended lower limit of 0.969. In both models, the regression weights indicated that all indicator variables loaded as significant factors and all group correlations were more than 0.5 which is the minimum recommended thus confirming a model fit for the three hope dimensions for the 10-item scale.

#### Validity of the 10-item hope scale-construct validity

At baseline, the mean of the 10 hope scale items was 2.6 (SD = 0.52, range 1.0–4.0, IQR = 0.46) at baseline. Pearson correlations coefficient between hope and our selected constructs were supportive of a priori hypotheses, although not all were significant. Higher scores of general knowledge about HIV had significant and positive correlation with the local hope scale at baseline (*r* = 0.281 *p* < 0.001) and at follow-up (*r* = 0.422, *p* < 0.001), similarly higher scores of knowledge about ART were significantly and positively correlated with hope although the Pearson coefficient was quite small at baseline (*r* = 0.161, *p* = 0.003) but high at follow-up (*r* = 0.544, *p* < 0.001). Likewise Self-esteem and social support, adherence to ART and attendance to scheduled clinic visits were also positively correlated with hope at baseline (*r* = 0.178, *p* < 0.001), (*r* = 0.209, *p* < 0.001), (*r* = 0.111, *p* = 0.001), and (*r* = 0.131, *p* = 0.0018) respectively. At 24 months of follow-up the same variables much high strength of positive correlations with hope except for social support and clinic visits which remained small but significant; Self-esteem (*r* = 0.449 *p* < 0.001), social support (*r* = 0.101 *p* = 0.003), attendance to scheduled clinic visits(*r* = 0.176 *p* = 0.04) and adherence to ART(*r* = 0.338 *p* < 0.001). The clinical outcome variables were also positively correlated with hope, and while this direction was confirmed the correlations were not significant at baseline; CD4 + T cell counts (*r* = 0.035, *p* = 0.532), hemoglobin (*r* = 0.061, *P* = 0.270) and Weight (*r* = 0.027, *p* = 0.626) and at 24 months follow-up the strength of the positive correlations increased but none of them were significantly correlated with hope; CD4 + T cell counts (*r* = 0.224, *p* = 0.0.09), hemoglobin (*r* = 0.118, *P* = 0.16) and Weight (*r* = 0.244, *p* = 0.07).

In terms of *divergent validity*, all the hypothesized variables correlated negatively with the local hope scale at both baseline and follow-up. Higher scores of depression measured using PHQ-9 tool and stigma had significant negative correlations with hope; (*r* = − 0.190, *p* < 0.001) and (*r* = − 0.276, *p* = 0.001) respectively at baseline and during follow-up same direction of correlation was observed but with much stronger correlations; depression (*r* = 0.556 *p* < 0.001) and stigma(*r* = 0.348 *p* < 0.01). In addition, the locally developed scales for depression *(sonona)* and stress (*msongo)* from populations of PLH had moderate to strong negative correlations with the local hope scale (*r* = − 0.411, *p* < 0.001) and (*r* = 0.398, *p* = 0.004) respectively at baseline and at follow-up the strength of negative correlation of *sonona* and with the local hope scale was much stronger (*r* = 0.566) while that of *msongo* decreased slightly (*r* = 0.310) but they were both significantly negatively correlated with hope (*p* < 0.001) (Table 3) and (See A1 of Additional file 1).

**Table 3:** Construct Validity Assessment: Pearson’s Correlation of Selected Variables with Mean Hope Scores (N = 326)

Validation measures	Baseline		24 Months follow up	
	Correlation with mean hope	*p*-value	Correlation with mean hope	*p*-value
Socio-demographic				
General HIV knowledge	0.281	< 0.001	0.422	< 0.001
Knowledge of ART medication	0.161	0.003	0.544	< 0.001
Mental health				
Depression PHQ9 scores(−)	−0.299	< 0.001	− 0.348	0.002
Self- esteem (+)	0.178	< 0.001	0.490	< 0.001
Stigma(s) (−)	−0.276	0.001	−0.446	< 0.001
Social support(+)	0.209	< 0.001	0.101	0.023
Local scale of stress *(msongo)*	−0.398	0.004	−0.310	< 0.001
Local scale of depression *(sonona)*	− 0.411	< 0.001	− 0.566	< 0.001
Behavioral risk				
Adherence to ART (+)	0.110	0.005	0.338	0.006
Attendance to clinic visits (+)	0. 131	0.018	0.176	0.040
Clinical outcomes				
CD4 (+)	0.035	0.532	0.224	0.092
Weight	0.027	0.626	0.244	0.070
Hemoglobin (BHG)	0.061	0.270	0.188	0.160

## Discussion

We have developed and validated a measure of hope which to our knowledge is the first to be developed for use specifically among adult PLH accessing care and treatment, in a high HIV prevalence urban setting of a low-income country. The three dimensions of hope in our scale are not completely different from previously developed hope scales. A more recently developed local hope scale among PLH in similar settings of South Africa, which focused on HIV infected young women aged 13–20 years [45], showed one dimension reflecting future expectations and meeting goals. This dimension is similar to the third dimension of our hope scale; positive future expectations and meeting goals. Moreover, our question items under this dimension were similar; Example our item HS23 “*I can reach/meet my goals*” was similar to the question item PM7 “*It is easy for me to reach my goals”* [45] in the South African local hope scale. Setting goals and working toward them effectively is an important aspect of hope. It is consistent with the most recent definition of hope which includes not just positive future expectations (optimism) but actually playing a role to establish the expected positive future [4, 72] by making and meeting goals. This dimension is also similar to that of the Snyder Hope Scale which has “goal-directed energy” as one of its dimensions of hope. The Herth Hope Scale however includes an item that explores the presence of goals [1, 64], though in our scale the three items on meeting goals, plans and positive future expectations loaded together as one dimension. This could mean that our current study participants associated goals and the pathway which is following plans in order meet goals for the expected positive future, similar to the above-mentioned argument about hope being optimistic about future and playing a role to get to the future.

The other dimension that we found in our local hope scale of positive emotions /affect. This is not reflected in many of the existing hope scales except the Herth hope scale where a dimension of positive readiness includes an item about a feeling of deep inner strength [64].

The third dimension of cognition about positive results of care as a dimension of hope is not reflected in any of the existing hope scales in similar settings and in developed countries. This could be due to the fact that our study population was receiving ART and had expressed positive beliefs on the effectiveness of ART as part of their hope attributions. Since ART does suppress HIV viral load to undetectable plasma levels allowing restoration of the immune system, most PLH with good adherence to ART would likely have no symptoms [73–75], making this population different from that of patients with other chronic or terminal illnesses that have been involved in the development of hope scales including persons with cancer, diabetes etc. Such patients may have disabling consequences of illness despite effective treatment/therapy compared to ART. It could also be a reflection of participant characteristics, as the majority (87%) of our participants were employed, self-employed, housewives and students who might be expecting to recover quickly from their symptoms in order to continue with their duties. A study that explored hope among patients with lung cancer reported that hope was inversely related to cancer symptom severity [76]. Persisting symptoms after starting ART may not be expected by PLH, explaining why this dimension may be important in this population. This new dimension could facilitate identification of clinical aspects of HIV care that if improved may prevent hopelessness and its negative impacts on mental health, adherence to ART and quality of life in PLH [28, 29]. It is also likely that this new dimension is reflecting the Tanzanian cultural values that relate with having no hope *mzigo* (which means becoming a burden or useless) to the relatives and family. As adults PLH with families, with some being widows and widowers, they value their ability to support their families. Likewise the PLH who are young strive to reach the culturally expected milestones of getting education, marriage within expected time, building a house and having children. Such achievements in Tanzanian culture brings a sense of honor, respect, dignity and motivation. Failing to this which can come as a result of HIV related frequent illnesses or prolonged poor health causes distress, frustration and hopelessness [77]. The fight to recover from symptoms and go back to work or proceed with school as normal could be a fight for survival need for the PLH to maintain their cultural values in this setting [12]. This is also similar to a study conducted in Afghanistan that found maintaining personal and social dignity based on cultural values was a key to hope [7].

However there are social structural issues that can also affect hope study of hope. This study focused on the hope scale development and psychometric properties however, social structural issues of hope including access to knowledge about HIV and available treatment, counseling, social support groups, support from families especially spouses, were discussed in our previous study before this [49]. Another study that explored the perceptions of hope among youths in Tanzania revealed that the street youths that attributed their hope to social structures including external attachments to supporting adults, shelter, money, ability to access schooling work and future. The youths in homes however attributed their hope to better school performances to enable them to take care of their parents and care takes in future [78]. This is similar to studies conducted in South African which found hope levels were different among marginalized groups and not different among age groups [79, 80].

These findings are similar to previous literature and supports the argument that the dimensions of hope include cultural context in which a person’s culture projects as hope or hopelessness and that hope is not a universal experience to all groups or only cognitive process of goal attainments, but contexts in which hope is attained including culture is important [81].

The local hope scale was developed considering the local cultural context, as attributions of hope were derived from the narratives of PLH while taking into consideration more globally understood dimensions of hope. The items in the local hope scale are internally consistent both within the overall scale and its three subscales (dimensions). Our narrative data from qualitative research availed many (40 items) potential items that informed the development of the local hope scale, with the three extracted dimensions having resonance in this population of PLH being collaborative influences from others, positive affect, spirituality and positive illness /treatment-related cognition, plans, goals and positive future expectations. Our Iterative factor reduction approach resulted in a 3-factor structure including affective/positive emotions, cognition of effective care and future optimism which were similar to what we saw in the qualitative data [45, 82]..

Our study found preliminary support of good construct validity of the hope scale in the directions and magnitude of correlations between hope and variables that we hypothesized to have correlation with hope. The strength of the correlation was moderate at baseline except for the clinical outcome variables of CD4, HBG and weight and local measures of depression and stress which were small but increased during follow-up which is attributed to the effect of *NAMWEZA* intervention. We also found convergent validity of the scale indicated by significant correlations of hope scale with depression, HIV knowledge, self-esteem, and social support, adherence to medications and clinic visits and clinical outcomes parameters.

This is similar to previous reports from multiple studies, that show hope was positively associated with knowledge [45], self-esteem [83, 84], social support [45], and adherence to medications and clinic visits [14–16]. Our study indicated that hope was not significantly correlated with clinical outcomes such as the CD4 positive T cell count, weight and Hemoglobin (HBG) levels. We found no previous literature linking the direct effects of hope on clinical outcomes. Similar to what we found, most literature reports that hope mediates [24] other factors including resilience, coping, compliance with care, self-esteem and quality of life which might impact clinical outcomes positively. The low rates of hopefulness found in this study population are perhaps characteristic of PLH accessing care as they were derived at baseline prior to the *NAMWEZA* psychosocial intervention that could likely improve hope.

The negative direction of correlation between hope and depression which indicated divergent validity was similar to what has been reported from previous studies [66]. A meta-analysis conducted in 2008 revealed that most studies reported hope was negatively correlated with the mental health disorders of depression and anxiety but positively correlated with subjective health, quality of life and self-esteem and [85]. A more recent study reported that hope was negatively correlated in persons with schizophrenia, with levels of internalized stigma, but positively correlated with resistance to stigma [86]. The hope scale was also negatively correlated with locally developed depression and stress scales at strengths that were mild to moderate when compared to the PHQ-9 depression scores, most likely because the local scales were developed in similar populations of PLH and in local culture thus the items may be clearer and have greater resonance. The almost similar strength of the correlations between the hope scale and our locally developed measures of stress and depression could also imply that these local distress terms used to describe depression and stress could have similar meanings as indicated in our previous study [49].

The hope measure could support the efforts to improve the wellbeing of PLH in high prevalent settings of Tanzania. Whereas studies have reported that preventing and addressing mental health concerns including anxiety and depression among PLH has the potential to improve HIV treatment outcomes [87], recent reports in Tanzania indicate both adolescents and adults living with HIV/AIDS, had increased rates of depression by four times in adolescents with compared to those without HIV [51, 88, 89]. In addition the integration of mental health care within the primary and HIV care services remains limited [43, 90–92] in part due to limited number of trained health care providers and even fewer with mental health care competencies as well as stigmatization of mental health conditions in the community [43, 90, 92, 93]. A meta-analysis of studies that report the effects of hope therapy indicated that while effects were small for management of particularly severe depression, hope theories can guide management of depression among patients with chronic illnesses [94]. Other studies supporting this observation show that hope therapy and other hope related interventions are effective in addressing depression among patients with chronic physical illnesses [21–23]. In this context, this simple measure for determining hope may be a first step to identifying clients for depression prevention interventions so as to improve HIV clinical outcomes the overall mental wellbeing and quality of life of PLH. Moreover interventions that improve access to ART comprehensive services, repeated counseling, managing side effects of ART [49, 77], and active monitoring of HIV viral load to ensure PLH are virally suppressed [53, 95–97] and thus healthy enough to able to return to their social and cultural priorities could increase hope and its other benefits among PLH.

## Limitations

The *NAMWEZA* study sample included only PLH enrolled in care and taking ART. This excluded PLH who were not yet enrolled in care and not taking ART. PLH in Tanzania who are not in care are more likely to have limited information about HIV, due to lack of frequent HIV care counseling offered in CTCs. They are also more likely to have deteriorating health due to the fact that they are not on ART thus their serum HIV viral load could to be high allowing them to get HIV related opportunistic infections. Excluding this group from this study potentially limits generalizability of the study to the PLH who are not yet in care or not taking ART.

Although the evaluation study happened in1 large hospital of Sinza which could limit generalizability, the tool was developed and piloted in 8 large CTCs across all districts of Dar es Salaam. The study was based on an urban congested setting with easier access to HIV care services in all districts. Rural settings, however, might have fewer HIV care clinics located a longer distance from homes, resulting in more difficulty for PLH to access HIV care services, which can impact the level of hope. Also our hope scale was not tested among people who were not HIV infected and we did not explore how the scale performed in different social groups like gender. These will be explored in future studies.

In addition our scale is much more inclined to issues of HIV/AIDS related symptoms and the ability to access clinical care, which can affect hope. This is because the scale is based on the perceptions of our population of PLH on hope in Tanzanian settings and the conceptual framework available in our previous paper which also include some social structural issues but their question items were removed from the scale because of poor loadings [49]. Being health was culturally very important because it allowed PLH to achieve their individual plans and what the community expected of them. However future studies exploring hope scales among PLH should explore other social structural issues.

## Conclusion

In summary, our study identified a locally derived 10-item latent measure of hope among adult men and women PLH in an area with a high prevalence of HIV. The extracted dimensions of the scale constructs were more than the common dimensions of goal oriented thinking, pathways and motivation previously reported in globally used measures of hope, but included positive cognition about HIV care received and its effectiveness mainly linked to becoming normal in order to support families. Evaluation of the local hope scale indicates it has demonstrated high construct validity and good reliability. This is a simple measure could support early identification and prevention of hopelessness and mild to moderate mental health conditions among PLH in Tanzania and similar settings.

## Supplementary information

**Additional file 1.** The value of hope: development and validation of a contextual measure of hope among people living with hiv in urban Dar es salaam, Tanzania.

## Data Availability

The data that support the findings of this study and the full trial protocol for this and for the main *NAMWEZA* trial on which this study is based, are available at the Management and Development for Health (MDH) central server *NAMWEZA* database file. Restrictions apply to the availability of these data, which were used under license for the current study, and so are not publicly available. Data are however available from the authors upon reasonable request through emails to authors and to MDH admin through mdh@mdh-tz.org.

## Abbreviations

ACASI: Audio Computer-Assisted Self-Interview
ART: Antiretroviral Therapy
CFA: Confirmatory Factor Analysis
CFI: Comparative Fit Index
CTC: Care and Treatment Clinic
EFA: Exploratory Factor Analysis
FGD: Focus Group Discussions
HBG: Hemoglobin
KI: Key Informants
PHQ-9: Patients Health questionnaire
RMSEA: Root Mean Square Error of Approximation
TLI: Tucker-Lewis Index
