# Supplementary material for: The value of hope: development and validation of a contextual measure of hope among people living with HIV in urban Tanzania a mixed methods exploratory sequential study
Source: BMC Psychol. 2020 Jan 29;8:5. doi: 10.1186/s40359-020-0376-y (PMC6988347; doi:10.1186/s40359-020-0376-y)
Supplement: Supplementary file 1 — Additional file 1. The value of hope: development and validation of a contextual measure of hope among people living with hiv in urban Dar es salaam, Tanzania. [file 40359_2020_376_MOESM1_ESM.pdf]

ADDITIONAL FILE 1

THE VALUE OF HOPE: DEVELOPMENT AND VALIDATION OF A CONTEXTUAL MEASURE OF HOPE AMONG PEOPLE LIVING WITH HIV IN URBAN DAR ES SALAAM, TANZANIA

Validation measures	Baseline		24 Months follow up	
	Predicted direction of association	Predicted magnitude of association	Predicted direction of association	Predicted magnitude of association
Socio-demographic				
General knowledge about HIV	Positive	Moderate	Positive	Strong
Knowledge of ART	Positive	Small to moderate	Positive	Moderate
Mental health				
Depression	Negative	Moderate	Negative	Moderate
Self-esteem	Positive	Mild	Positive	strong
Stigma	Negative	Moderate	Negative	Moderate-strong
Local scale of stress(*msongo*)	Negative	Mild to Moderate	Negative	Moderate to strong
Local scale of depression(*sonona*)	Negative	Moderate to strong	Negative	Moderate to strong
Overall social support	Positive	Moderate to strong	Positive	Moderate to strong
Behavioral risk				
Adherence to ART	Positive	Small to moderate	Positive	Moderate to strong
Attending clinic	Positive	Small to moderate	Positive	Moderate to strong
Clinical outcomes				
CD4 cell counts	Positive	Small to moderate	Positive	Moderate to strong
Weight	Positive	Small to moderate	Positive	Small to moderate

A1; Direction and magnitude of hypothesized relationships between validation variables and hope at baseline and follow up

ITEM NUMBER	ITEMS
1.	I have new strength (AGENCY/READINESS TO TAKE ACTION)
2.	I feel uplifted (AFFECTIVE/SPIRITUAL)
3.	I feel comforted (AFFECTIVE/SPIRITUAL/SOCIAL SUPPORT)
4.	My heart is cheerful (AFFECTIVE/SPIRITUAL)
5.	I feel encouraged (AFFECTIVE/SPIRITUAL/SOCIAL SUPPORT)
6.	I have peace (AFFECTIVE/SPIRITUAL)
7.	I have a lot of strength (COGNITIVE)
8.	I prefer meeting with other PLH than to be alone at home (AFFILIATIVE-CONTEXTUAL)
9.	I believe in my future plans (COGNITIVE PLANING)
10.	All my needs for care of my HIV condition are met (COGNITIVE/SATISFACTION WITH CARE)
11.	I can meet my goals (COGNITIVE TEMPORAL/ FUTURE ANTICIPATION)
12.	I have plans that control/lead my life (AFFECTIVE-BEHAVIORAL/GOAL SETTINGS
13.	I have the ability to take care of my health (COGNITIVE)
14.	I think about plans I have ahead of me (AFFECTIVE-BEHAVIORAL/ FUTURE ANTICIPATION) Revised to I think about my future plans
15.	I always get someone who talks to me well when I have lost hope(INFLUENCE OF OTHERS/AFFILIATIVE)
16.	I feel HIV will kill me, is like a death sentence(COGNITIVE/HIV SPECIFIC)
17.	I have authority over my life (COGNITIVE /ESCAPE FROM DESPAIR)
18.	I can freely say to anyone HIV is my real situation and I have accepted it(COGNITIVE/HIV SPECIFIC)
19.	My spouse encourages me when I am discouraged(ROLE OF OTHERS/AFFILIATIVE)
20.	I usually have someone to talk to when I face a problem(ROLE OF OTHERS/AFFILIATIVE)
21.	I was able to test for HIV, receive my results and understood my results of HIV positive diagnosis(COGNITIVE/HIV SPECIFIC)
22.	I feel good when something bad I anticipated didn't happen(AFFECTIVE/ESCAPING DESPAIR/positive expectation)
23.	Personally, I am self-aware of my HIV infection situation(COGNITIVE/ HIV SPECIFIC)
24.	I have accepted my HIV diagnosis(COGNITIVE/HIV SPECIFIC)
25.	My parents encourage me when I lose hope (INFLUENCE OF OTHERS/AFFILIATIVE)
26.	My relatives encourage me when I have lost hope (INFLUENCE OF OTHERS/AFFILIATIVE)
27.	I can live long and continue with life activities after HIV diagnosis(COGNITIVE/ESCAPE FROM DESPAIR/OPTIMISM)
28.	I feel I have improved since I started ARVs(AFFECTIVE/ESCAPE FROM DESPAIR/HIV CARE/effectiveness of HIV care)
29.	I am satisfied with my progress since I started ARVs(AFFECTIVE/ HIV CARE/ESCAPE FROM DESPAIR)
30.	As I do what my counselor tells me, I am satisfied with the results I see (COGNITIVE/ESCAPE FROM DESPAIR)
31.	I get ideas from other people which give me strength (INFLUENCE OF OTHERS/AFFILIATIVE)
32.	I am Happy (AFFECTIVE)
33.	I feel good that I tested for HIV, understood and accepted the results (The effect of an HIV diagnosis) (revised) I have accepted my HIV diagnosis(AFFECTIVE /ESCAPE FROM DISPAIR)
34.	I feel I have new energy (AFFECTIVE/URGENCY /DESIRE TO TAKE ACTION)
35.	I can have children even when I am HIV positive(COGNITIVE /OPTIMISM)

36.	Knowing my HIV status gives me relief (COGNITIVE/HIV SPECIFIC)
37.	My family encourages me when I am discouraged(INFLUENCE OF OTHERS/AFFILIATIVE)
38.	Testing for HIV and understanding and accepting the results made me hopeful(COGNITIVE HIV SPECIFIC)
39.	I believe the good thing I anticipate will happen (COGNITIVE/POSITIVE ANTICIPATION)
40.	I live with clean health I don't have illnesses in my body (POSITIVE ANTICIPATION

A 2: Initial 40 items grouped in broader dimensions and items from qualitative data and 1st expert opinion and rate in a scale of 1-5

DOMAIN I: *POSITIVE EMOTIONS /FEELINGS/REACTION/SPIRITUAL/FAITH*

1. I feel uplifted
2. My heart is cheerful
3. I feel comforted
4. I feel encouraged
5. I have peace
6. I feel I am joyful
7. I feel I have new strength
8. I feel I have a lot of strength
9. I feel I have new energy

DOMAIN II: *CURRENT AND FUTURE WORRIES/NEGATIVE ATTITUDES OF LIVING WITH HIV*

10. I feel HIV will kill me, is like a death sentence

DOMAIN III*: SATISFACTION WITH CARE/ POSITIVE ATTITUDES OF LIVING WITH HIV*

11. I feel I have improved since I started ARVs
12. I am satisfied with my progress since I started ARVs
13. Knowing my HIV status gives me relief
14. Testing for HIV, understanding and accepting the results made he hopeful
15. I was able to test for HIV, received my results and understood my results of HIV positive diagnosis
16. I live with clean health I don't have illnesses in my body
17. I have the ability to take care of my health
18. I can freely say to anyone HIV is my real situation and I have accepted it
19. As I do what my counselor/health worker tells me I am satisfied with the results I see
20. Personally, I am self-conscious about my HIV condition, I have accepted it
21. All my needs for care of my HIV condition are met
22. I feel good that I tested for HIV, understood and accepted the results
23. I have accepted my HIV diagnosis

DOMAIN IV: *PLANNING/PLANS AND GOALS*

24. I believe in my plans
25. I have authority over my life
26. I can meet my goals
27. I have plans that control/lead my life
28. I can meet my goals

DOMAIN V: *POSITIVE FUTURE EXPECTATION*

29. I feel good when something bad I anticipated did not happen
30. I think about the plans that I have ahead of me
31. I can have children even when I have HIV
32. I believe the good things I anticipate will happen
33. I can live long and continue with my life activities after HIV diagnosis

DOMAIN VI: *SUPPORT FROM OTHERS*

34. I always get someone who talks to me well when I have lost hope
35. I prefer meeting with other PLH than to be alone at home
36. My spouse encourages me when I am discouraged
37. My relatives encourage me when I have lost hope
38. My parents encourage me when I have lost hope
39. I usually have someone to talk to when I am faced with a problem
40. I get ideas from other people which gives me strength

A3: Summary of the domains and specific hope items identified in pile sorting exercise by PLH

ITEM NUMBER	ITEM REMOVED	ITEM MEASURING SAME/SIMILAR THING REPLACED BY
1.	I feel I have new energy	Item 7,1
2.	I can have children even when I am HIV positive	Item 27
3.	Knowing my HIV status gives me relief	Items 33, 21 and 24
4.	My family encourages me when I am discouraged	Items 19, 25 and 26
5.	Testing for HIV and understanding and accepting the results made me hopeful	Items 21, 24 and 33
6.	I believe what I anticipate will happen	Item 22
7.	I live with clean health I don’t have illnesses in my body	Item 27

A 4: list of items removed after the 2nd expert opinion

Item #	Item	Definitely False n (%)	Somewhat False n (%)	Somewhat True n (%)	Definitely True n (%)
1.	I feel I have a lot of strength	32 (10.1)	69 (21.7)	135 (42.5)	82 (25.8)
2.	I feel uplifted in my heart	32 (10.1)	47 (14.8)	137 (43.1)	102 (32.1)
3.	I have peace	13 (4.1)	33 (10.4)	104 (32.7)	168 (52.8)
4.	I feel I have new strength	32 (10.1)	69 (21.7)	135 (42.5)	82 (25.8)
5.	I am joyful	16 (5.0)	41 (12.9)	84 (26.4)	177 (55.7)
6.	My heart is cheerful	17 (5.3)	57 (17.9)	90 (28.3)	154 (48.4)
7.	All my needs for HIV care that I need for my HIV condition are all met	15 (4.7)	55 (17.3)	124 (39.0)	124 (39.0)
8.	I feel comforted	12 (3.8)	53 (16.3)	108 (34.0)	145 (45.6)
9.	The information I received from other people on how to solve my problems give me strength	38 (11.9)	48 (15.1)	120 (37.7)	112 (35.2)
10.	I always have someone to talk to me well when I have lost hope	45 (14.5)	48 (15.1)	101 (31.8)	124 (39.0)
11.	Husband/wife /my life partner encourages me when I have lost hope	77 (24.2)	43 (13.5)	96 (30.2)	102 (32.1)
12.	I usually have someone to talk to when I have a problem	45 (14.2)	48 (15.1)	101 (31.8)	124 (39.0)
13.	My parents encourage me when I lose hope	67 (21.1)	48 (15.1)	93 (29.2)	110 (34.6)
14.	My relatives encourage me when I have lost hope	50 (15.7)	56 (17.6)	96 (30.5)	115 (36.2)
15.	Personally, I am aware/self-aware of my HIV infection situation	7 (2.2)	25 (7.9)	93 (29.3)	193 (60.7)
16.	I feel relieved /relief by testing and knowing my HIV diagnosis	25(7.9)	69 (21.7)	103 (32.4)	121 (38.1)
17.	I prefer meeting with other people living with HIV rather than sitting alone at home	32 (10.1)	43 (13.5)	98 (30.8)	145 (45.6)
18.	I am free to tell anyone that I am HIV positive	141 (44.3)	67 (21.1)	51 (16.0)	59 (18.6)
19.	I feel I have improved since I started ART	16 (5.0)	43(13.5)	108(34.0)	151 (47.5)
20.	I am satisfied with my progress since I started ARVs	21 (6.6)	24(7.5)	128(40.3)	145 (45.6)
21.	I believe to follow the advice from my counselor, brings me good results for my health	6 (1.9)	18(5.7)	115(36.2)	179 (56.3)
22.	I have authority over my life	10 (3.1)	43 (13.5)	100 (37.1)	171 (53.8)
23.	I believe in my plans	13 (4.3)	43 (13.5)	109 (34.3)	153 (48.1)
24.	I can reach my goals	13 (9.7)	41 (12.9)	109 (34.3)	137 (43.1)
25.	I can live a long life and continue with my routine activities	29 (9.1)	47 (14.8)	106 (33.3)	136 (42.8)
26.	To have HIV is like a death sentence	162 (50.9)	51 (16.0)	50 (15.7)	55 (17.3)
27.	I feel good if something bad that I had anticipated didn't happen	35 (11)	76 (23.9)	78 (24.5)	129 (40.6)
28.	I feel encouraged	11 (3.5)	39 (12.3)	123 (38.7)	145 (45.6)
29.	I have the ability to take care of my health	1 (2.2)	25 (7.9)	146 (45.9)	140 (44.0)
30.	*I was able to test for HIV, receive my results and understood my results of HIV positive diagnosis	1 (0.3)	0 (0.0)	2 (0.6)	10 (3.1)
31.	I think about my future plans	13 (4.1)	43 (13.5)	125 (39.3)	137 (43.1)
32.	I have plans which lead/guide my life/plans for my life	17 (5.3)	60 (18.9)	123 (38.7)	118 (37.1)
33.	I have accepted my HIV condition	14 (4.4)	15 (4.7)	76 (23.9)	213 (67.0)

A 5: Percent distribution of hope scale items responses during the pilot

ITEM	BASELINE					FOLLOW UP				
	Scale Mean if Item Deleted	Scale Variance if Item Deleted	Total Correlation	Squared Multiple Correlation	Cronbach's Alpha if Item Deleted	Scale Mean if Item Deleted	Scale Variance if Item Deleted	Total Correlation	Squared Multiple Correlation	Cronbach's Alpha if Item Deleted
I have new strength	29.02	28.13	0.61	0.48	0.85	32.43	23.52	0.93	0.92	0.97
I am Happy	29.18	28.32	0.54	0.43	0.85	32.44	23.42	0.94	0.96	0.97
I have piece	29.03	27.83	0.59	0.50	0.85	32.45	23.55	0.91	0.93	0.97
My heart is cheerful	29.16	27.43	0.61	0.47	0.85	32.44	23.44	0.94	0.94	0.97
I am satisfied with my progress since I started ART	29.24	28.97	0.48	0.32	0.86	32.44	24.86	0.71	0.71	0.97
I believe to follow the advice from my counselor brings me good results for health	29.11	27.98	0.60	0.53	0.85	32.43	24.27	0.85	0.90	0.97
All my needs for HIV Care and Treatment have been met	28.89	29.09	0.61	0.53	0.85	32.44	24.35	0.83	0.86	0.97
I believe in my plans	29.10	28.04	0.60	0.49	0.85	32.41	24.14	0.89	0.90	0.97
I think I can reach my goals	29.25	26.68	0.65	0.52	0.84	32.42	24.27	0.85	0.89	0.97
I can live a long life and continue with my routine activities	29.26	28.20	0.49	0.35	0.86	32.40	24.49	0.80	0.79	0.97

A6: Total Items statistics for the local hope scale at baseline and at 24 months
